# International Society of Sports Nutrition position stand: protein and exercise

**DOI:** 10.1186/1550-2783-4-8

**Published:** 2007-09-26

**Authors:** Bill Campbell, Richard B Kreider, Tim Ziegenfuss, Paul La Bounty, Mike Roberts, Darren Burke, Jamie Landis, Hector Lopez, Jose Antonio

**Affiliations:** 1 Exercise and Performance Nutrition Laboratory, Dept. of Physical Education and Exercise Science, University of South Florida, 4202 E. Fowler Avenue, PED 214, Tampa, FL 33620, USA; 2 Exercise and Sport Nutrition Laboratory, Dept. of Health, Human Performance, and Recreation, Baylor University, One Bear Place 97313, Waco, TX 76798-7313, USA; 3 Ohio Research Group of Exercise Science & Sports Nutrition, Wadsworth Medical Center, 323 High St, STE 103A, Wadsworth, OH 44281, USA; 4 Exercise and Sport Nutrition Laboratory, Dept. of Health, Human Performance, and Recreation, Baylor University, One Bear Place 97313, Waco, TX 76798-7313, USA; 5 Applied Biochemistry and Molecular Physiology Laboratory, Department of Health and Exercise Science, University of Oklahoma, 1401 Asp Avenue, Norman, OK 73019, USA; 6 Exercise Science Laboratory, Dept. of Human Kinetics, St. Francis Xavier University, P.O. Box 5000 Antigonish, Nova Scotia, B2G 2W5, Canada; 7 Department of Biology, Lakeland Community College, 7700 Clocktower Drive, Kirtland, Ohio 44094-5198, USA; 8 Northwestern University Feinberg School of Medicine, Department of Physical Medicine and Rehabilitation, Rehabilitation Institute of Chicago, 345 East Superior Street, Chicago, IL 60611, USA; 9 Department of Exercise Science and Health Promotion, Florida Atlantic University, 2912 College Avenue, Davie, FL 33314, USA

## Abstract

**Position Statement:**

The following seven points related to the intake of protein for healthy, exercising individuals constitute the position stand of the Society. They have been approved by the Research Committee of the Society. 1) Vast research supports the contention that individuals engaged in regular exercise training require more dietary protein than sedentary individuals. 2) Protein intakes of 1.4 – 2.0 g/kg/day for physically active individuals is not only safe, but may improve the training adaptations to exercise training. 3) When part of a balanced, nutrient-dense diet, protein intakes at this level are not detrimental to kidney function or bone metabolism in healthy, active persons. 4) While it is possible for physically active individuals to obtain their daily protein requirements through a varied, regular diet, supplemental protein in various forms are a practical way of ensuring adequate and quality protein intake for athletes. 5) Different types and quality of protein can affect amino acid bioavailability following protein supplementation. The superiority of one protein type over another in terms of optimizing recovery and/or training adaptations remains to be convincingly demonstrated. 6) Appropriately timed protein intake is an important component of an overall exercise training program, essential for proper recovery, immune function, and the growth and maintenance of lean body mass. 7) Under certain circumstances, specific amino acid supplements, such as branched-chain amino acids (BCAA's), may improve exercise performance and recovery from exercise.

## Protein intake recommendations

Controversy has existed over the safety and effectiveness of protein intake above that currently recommended. Currently, the RDA for protein in healthy adults is 0.8 g/kg body weight per day [1]. The purpose of this recommendation was to account for individual differences in protein metabolism, variations in the biological value of protein, and nitrogen losses in the urine and feces. Many factors need to be considered when determining an optimal amount of dietary protein for exercising individuals. These factors include protein quality, energy intake, carbohydrate intake, mode and intensity of exercise, and the timing of the protein intake [2]. The current recommended level of protein intake (0.8 g/kg/day) is estimated to be sufficient to meet the need of nearly all (97.5%) healthy men and women age 19 years and older. This amount of protein intake may be appropriate for non-exercising individuals, but it is likely not sufficient to offset the oxidation of protein/amino acids during exercise (approximately 1–5% of the total energy cost of exercise) nor is it sufficient to provide substrate for lean tissue accretion or for the repair of exercise induced muscle damage [3,4].

Protein recommendations are based upon nitrogen balance assessment and amino acid tracer studies. The nitrogen balance technique involves quantifying the total amount of dietary protein that enters the body and the total amount of the nitrogen that is excreted [5]. Nitrogen balance studies may underestimate the amount of protein required for optimal function because these studies do not directly relate to exercise performance. Also, it is possible that protein intake above those levels deemed necessary by nitrogen balance studies may improve exercise performance by enhancing energy utilization or stimulating increases in fat-free mass in exercising individuals [2]. Indeed, an abundance of research indicates that those individuals who engage in physical activity/exercise require higher levels of protein intake than 0.8 g/kg body weight per day, regardless of the mode of exercise (i.e. endurance, resistance, etc.) or training state (i.e. recreational, moderately or well-trained) [6-13]. Also, there is a genuine risk in consuming insufficient amounts of protein, especially in the context of exercise; a negative nitrogen balance will likely be created, leading to increased catabolism and impaired recovery from exercise [14].

Relative to endurance exercise, recommended protein intakes range from of 1.0 g/kg to 1.6 g/kg per day [2,4,7,15] depending on the intensity and duration of the endurance exercise, as well as the training status of the individual. For example, an elite endurance athlete requires a greater level of protein intake approaching the higher end the aforementioned range (1.0 to 1.6 g/kg/day). Additionally, as endurance exercise increases in intensity and duration, there is an increased oxidation of branched-chain amino acids, which creates a demand within the body for protein intakes at the upper end of this range. Strength/power exercise is thought to increase protein requirements even more than endurance exercise, particularly during the initial stages of training and/or sharp increases in volume. Recommendations for strength/power exercise typically range from 1.6 to 2.0 g/kg/day [3,11-13,16], although some research suggests that protein requirements may actually decrease during training due to biological adaptations that improve net protein retention [17].

Little research has been conducted on exercise activities that are intermittent in nature (e.g., soccer, basketball, mixed martial arts, etc.). In a review focusing on soccer players, a protein intake of 1.4–1.7 g/kg was recommended [18]. Protein intakes within this range (1.4 to 1.7 g/kg/day) are recommended for those engaging in other types of intermittent sports.

In summary, it is the position of the International Society of Sport Nutrition that exercising individuals ingest protein ranging from 1.4 to 2.0 g/kg/day. Individuals engaging in endurance exercise should ingest levels at the lower end of this range, individuals engaging in intermittent activities should ingest levels in the middle of this range, and those engaging in strength/power exercise should ingest levels at the upper end of this range.

## Safety of protein intakes higher than RDA

It is often erroneously reported by popular media that a chronically high protein intake is unhealthy and may result in unnecessary metabolic strain on the kidneys leading to impaired renal function. Another concern that is often cited is that high protein diets increase the excretion of calcium thereby increasing the risk for osteoporosis. Both of these concerns are unfounded as there is no substantive evidence that protein intakes in the ranges suggested above will have adverse effects in healthy, exercising individuals.

One of the main points of debate relative to protein intake and kidney function is the belief that habitual protein consumption in excess of the RDA promotes chronic renal disease through increased glomerular pressure and hyperfiltration [19,20]. The majority of scientific evidence cited by the authors [20] was generated from animal models and patients with co-existing renal disease. As such, the extension of this relationship to healthy individuals with normal renal function is inappropriate [21]. In a well designed prospective cohort study, it was surmised that high protein intake was not associated with renal functional decline in women with normally operating kidneys [22]. Also, it has been reported that there are no statistically significant differences in age, sex, weight, and kidney function between non-vegetarians and vegetarians (a group demonstrated to have lower dietary protein intakes) [23,24]. Both the non-vegetarian and vegetarian groups possessed similar kidney function, and displayed the same rate of progressive deterioration in renal physiology with age [24]. Preliminary clinical and epidemiological studies have suggested a benefit of relatively high protein diets on major risk factors for chronic kidney disease, such as hypertension, diabetes, obesity and metabolic syndrome. Future studies are necessary to further examine the role of relatively high protein weight loss diets, dietary protein source (quality) and quantity on the prevalence and development of kidney disease in at risk patient populations [25,26]. While it appears that dietary protein intakes above the RDA are not deleterious for healthy, exercising individuals, those individuals with mild renal insufficiency need to closely monitor their protein intake as observational data from epidemiological studies provide evidence that dietary protein intake may be related to the progression of renal disease [21,26].

In addition to renal function, the relationship between dietary protein intake and bone metabolism has also served as the cause for some controversy. Specifically, there is concern that a high intake of dietary protein results in the leaching of calcium from bones, which may lead to osteopenia and predispose some individuals to osteoporosis. This supposition stems from early studies reporting an increase in urine acidity from increased dietary protein that appeared to be linked to drawing calcium from the bones to buffer the acid load. However, studies reporting this effect were limited by small sample sizes, methodological errors, and the use of high doses of purified forms of protein [27]. It is now known that the phosphate content of protein foods (and supplements fortified with calcium and phosphorous) negates this effect. In fact, some data suggest that elderly men and women (the segment of the population most susceptible to osteoporosis) should consume dietary protein above current recommendations (0.8 g/kg/day) to optimize bone mass [28]. In addition, data from stable calcium isotope studies is emerging, which suggests the main source of the increase in urinary calcium from a high-protein diet is intestinal (dietary) and not from bone resorption [29]. Also, given that exercise training supplies the stimulus for increasing skeletal muscle protein, levels in the range of 1.4 to 2.0 g/kg/d are recommended to transform this stimulus into additional contractile tissue, which is an important predictor in bone mass accrual during pre-pubertal growth [30,31]. More research needs to be conducted in adults and the elderly relative to exercise, skeletal muscle hypertrophy and protein intake and their cumulative effects on bone mass. Overall, there is a lack of scientific evidence linking higher dietary protein intakes to adverse outcomes in healthy, exercising individuals. There is, however, a body of scientific literature which has documented a benefit of protein supplementation to the health of multiple organ systems. It is therefore the position of the International Society of Sport Nutrition that active elderly individuals require protein intakes ranging from 1.4 to 2.0 g/kg/day, and that this level of intake is safe.

## Protein quality and common types of protein supplements

To obtain supplemental dietary protein, exercising individuals often ingest protein powders. Powdered protein is convenient and, depending on the product, can be cost-efficient as well [32]. Common sources of protein include milk, whey, casein, egg, and soy-based powders. Different protein sources and purification methods may affect the bioavailability of amino acids. The amino acid bioavailability of a protein source is best conceptualized as the amount and variety of amino acids that are digested and absorbed into the bloodstream after a protein is ingested. Furthermore, amino acid bioavailability may also be reflected by the difference between the nitrogen content from a protein source that is ingested versus the nitrogen content that is subsequently present in the feces. Consideration of the bioavailability of amino acids into the blood, as well as their delivery to the target tissue(s), is of greatest importance when planning a regimen of pre- and post-exercise protein ingestion. A protein that provides an adequate circulating pool of amino acids before and after exercise is readily taken up by skeletal muscle to optimize nitrogen balance and muscle protein kinetics [33].

The quality of a protein source has previously been determined by the somewhat outdated protein efficiency ratio (PER), and the more precise protein digestibility corrected amino acid score (PDCAAS). The former method was used to evaluate the quality of a protein source by quantifying the amount of body mass maturing rats accrue when fed a test protein. The latter method was established by the Food and Agriculture Organization (FAO 1991) as a more appropriate scoring method which utilized the amino acid composition of a test protein relative to a reference amino acid pattern, which was then corrected for differences in protein digestibility [34]. The U.S. Dairy Export Council's Reference Manual for U.S. Whey and Lactose Products (2003) indicates that milk-derived whey protein isolate presents the highest PDCAAS out of all of the common protein sources due to its high content of essential and branched chain amino acids. Milk-derived casein, egg white powder, and soy protein isolate are also classified as high quality protein sources with all of them scoring a value of unity (1.00) on the PDCAAS scale. In contrast, lentils score a value of 0.52 while wheat gluten scores a meager 0.25.

Commercially, the two most popular types of proteins in supplemental form are whey and casein. Recent investigations have detailed the serum amino acid responses to ingesting different protein types. Using amino acid tracer methodology, it was demonstrated that whey protein elicits a sharp, rapid increase of plasma amino acids following ingestion, while the consumption of casein induces a moderate, prolonged increase in plasma amino acids that was sustained over a 7-hr postprandial time period [35]. The differences in the digestibility and absorption of these protein types may indicate that the ingestion of "slow" (casein) and "fast" (whey) proteins differentially mediate whole body protein metabolism due to their digestive properties [35]. Other studies have shown similar differences in the peak plasma levels of amino acids following ingestion of whey and casein fractions (i.e., whey fractions peaking earlier than casein fractions) [36,37].

Applied exercise science research has also demonstrated the differential effects that ingesting different proteins exerts on postprandial blood amino acid responses and muscle protein synthesis after exercise. The data are equivocal relative to which type of protein increases net protein status (breakdown minus synthesis) to a greater extent after exercise. Some research has demonstrated that despite different patterns of blood amino acid responses, muscle protein net balance was similar in those ingesting casein or whey [33]. However, additional research has indicated that whey protein induced protein gain to a greater extent than casein [38]. In contrast, several other studies have shown that casein increased protein deposition at levels greater than whey proteins [35,37].

The recommendation of the International Society of Sport Nutrition is that individuals engaging in exercise attempt to obtain their protein requirements through whole foods. When supplements are ingested, we recommend that the protein contain both whey and casein components due to their high protein digestibility corrected amino acid score and ability to increase muscle protein accretion.

## Protein timing

It is generally recognized that active individuals require more dietary protein due to an increase in intramuscular protein oxidation [39] and protein breakdown [40] that occurs during exercise, as well as the need to further complement intramuscular protein resynthesis and attenuate proteolytic mechanisms that occur during the post-exercise recovery phases [41-43]. Thus, a strategically planned protein intake regimen timed around physical activity is integral in preserving muscle mass or eliciting muscular hypertrophy, ensuring a proper recovery from exercise, and perhaps even sustaining optimal immune function. Previously, high levels of blood amino acids following a bout of resistance training have been found to be integral in promoting muscle protein synthesis [44]. Evidence is accumulating that supports the benefits of the timing of protein intake and its effect on gains in lean mass during resistance exercise training [45-49]. Given that much of the research to date has been conducted on resistance exercise, more investigations are required to ascertain the effects of protein timing on other modes of exercise.

Research has also highlighted the positive immune and health-related effects associated with post-exercise protein ingestion. A previous investigation utilizing 130 United States Marine subjects [50] examined the effects of an ingested supplement (8 g carbohydrate, 10 g protein, 3 g fat) immediately after exercise on the status of various health markers. These data were compared to 129 subjects ingesting a non-protein supplement (8 g carbohydrate, 0 g protein, 3 g fat), and 128 subjects ingesting placebo tablets (0 g carbohydrate, 0 g protein, 0 g fat). Upon the completion of the 54-d trial, researchers reported that the subjects ingesting the protein supplement had an average of 33% fewer total medical visits, including 28% less visits due to bacterial or viral infections, 37% less orthopedic-related visits, and 83% less visits due to heat exhaustion. Moreover, post-exercise muscle soreness was significantly reduced in subjects ingesting protein when compared to the control groups. Previous studies using animal models have demonstrated that whey protein elicits immuno-enhancing properties, likely due to its high content of cysteine; an amino acid that is needed for glutathione production [51,52]. Hence, previous research has indicated that ingesting a protein source that is rich in essential amino acids and is readily digestible immediately before and following exercise training is beneficial for increasing muscle mass, recovery following exercise, and sustaining immune function during high-volume training periods. While protein ingestion is emphasized in this article, the concomitant ingestion of protein and carbohydrates prior to and/or following exercise has also been shown to be advantageous in increasing muscle protein synthesis; a result which is likely due to an increase in insulin signaling following the ingestion of carbohydrates.

It is the position of the International Society of Sport Nutrition that exercising individuals should consume high quality protein within the time period encompassing their exercise session (i.e. before, during, and after).

## The role of BCAA's in exercise

The branched-chain amino acids (i.e. leucine, isoleucine and valine) constitute approximately one-third of skeletal muscle protein [53]. An increasing amount of literature suggests that of the three BCAAs, leucine appears to play the most significant role in stimulating protein synthesis [54]. In this regard, amino acid supplementation (particularly the BCAAs) may be advantageous for the exercising individual.

A few studies reported that when BCAAs were infused in humans at rest, protein balance increases by either decreasing the rate of protein breakdown, increasing the rate of protein synthesis or a combination of both [55,56]. Following resistance exercise in males it has been shown that the addition of free leucine combined with carbohydrate and protein led to a greater increase protein synthesis as compared to taking the same amount of carbohydrate and protein without leucine [57]. However, the majority of the research relative to leucine ingestion and protein synthesis has been conducted using animal models. Similar research needs to be conducted in healthy individuals engaging in resistance exercise.

BCAA ingestion has been shown to be beneficial during aerobic exercise. When BCAAs are taken during aerobic exercise the net rate of protein degradation has been shown to decrease [58]. Equally important, BCAA administration given before and during exhaustive aerobic exercise to individuals with reduced muscle glycogen stores may also delay muscle glycogen depletion [59]. When BCAAs were given to runners during a marathon it improved the performance of "slower" runners (those who completed the race in 3.05 h-3.30 h) as compared to "faster" runners (those who completed the race in less than 3.05 h) [60]. Although there are numerous reported metabolic causes of fatigue such as glycogen depletion, proton accumulation, decreases in phosphocreatine levels, hypoglycemia, and increased free tryptophan/BCAA ratio, it is the increase in the free tryptophan/BCAA ratio that may be attenuated with BCAA supplementation. During prolonged aerobic exercise, the concentration of free tryptophan increases and the uptake of tryptophan into the brain increases. When this occurs, 5-hydroxytryptamine (a.k.a. serotonin), which is thought to play a role in the subjective feelings of fatigue, is produced. Similarly, BCAAs are transported into the brain by the same carrier system as tryptophan and thus "compete" with tryptophan to be transported into the brain. Therefore, it is believed that when certain amino acids such as BCAAs are present in the plasma in sufficient amounts, it theoretically may decrease the uptake of tryptophan in the brain and ultimately decrease the feelings of fatigue [61,62].

Furthermore, there is also research to suggest that BCAA administration taken during prolonged endurance events may help with mental performance in addition to the aforementioned performance benefits [60]. However, not all research investigating BCAA supplementation has reported improvements in exercise performance. One such study [63] reported that leucine ingestion taken before and during anaerobic running to exhaustion (200 mg/kg of body weight) and during a strength training session (100 mg/kg of body weight) did not improve exercise performance. Reasons for discrepant results are not clear at this time, but at the very minimum, it seems apparent that supplementation with BCAAs does not impair performance.

Because BCAAs have been shown to aid in recovery processes from exercise such as stimulating protein synthesis, aiding in glycogen resynthesis, as well as delaying the onset of fatigue and helping maintain mental function in aerobic-based exercise, we suggest consuming BCAAs (in addition to carbohydrates) before, during, and following an exercise bout. It has been suggested that the RDA for leucine alone should be 45 mg/kg/day for sedentary individuals, and even higher for active individuals [53]. However, while more research is indicated, because BCAAs occur in nature (i.e. animal protein) in a 2:1:1 ratio (leucine: isoleucine: valine), one may consider ingesting ≥ 45 mg/kg/day of leucine along with approximately ≥ 22.5 mg/kg/day of both isoleucine and valine in a 24 hour time frame in order to optimize overall training adaptations. This will ensure the 2:1:1 ratio that appears often in animal protein [64]. It should not be overlooked that complete proteins in whole foods, as well as most quality protein powders, contain approximately 25% BCAAs. Any deficiency in BCAA intake from whole foods can easily be remedied by consuming whey protein during the time frame encompassing the exercise session; however, an attempt should be made to obtain all recommended BCAAs from whole food protein sources.

## Conclusion

It is the position of the International Society of Sports Nutrition that exercising individuals need approximately 1.4 to 2.0 grams of protein per kilogram of bodyweight per day. The amount is dependent upon the mode and intensity of the exercise, the quality of the protein ingested, and the status of the energy and carbohydrate intake of the individual. Concerns that protein intake within this range is unhealthy are unfounded in healthy, exercising individuals. An attempt should be made to obtain protein requirements from whole foods, but supplemental protein is a safe and convenient method of ingesting high quality dietary protein. The timing of protein intake in the time period encompassing the exercise session has several benefits including improved recovery and greater gains in fat free mass. Protein residues such as branched chain amino acids have been shown to be beneficial for the exercising individual, including increasing the rates of protein synthesis, decreasing the rate of protein degradation, and possibly aiding in recovery from exercise. In summary, exercising individuals need more dietary protein than their sedentary counterparts, which can be obtained from whole foods as well as from high quality supplemental protein sources such as whey and casein protein.

## Abbreviations

g/kg/d = grams per kilogram of bodyweight per day

BCAAs = branched-chain amino acids

## Competing interests

The author(s) declare that they have no competing interests.
